# Employment, education, and family: Revealing the motives behind internal migration in Great Britain

**DOI:** 10.1002/psp.2233

**Published:** 2019-04-10

**Authors:** Michael J. Thomas

**Affiliations:** ^1^ Population Research Centre, Faculty of Spatial Sciences University of Groningen Groningen The Netherlands

**Keywords:** family migration, Great Britain, internal migration, migration distance, migration motives, UKHLS

## Abstract

Distinctions between internal migration and residential mobility are often formed with reference to assumed differences in motivation, with migration typically linked to employment and educational motives and shorter distance mobility to housing and family. Using geocoded microdata, this article reveals how employment‐led migration represents only a minority share (≈30%) of total migration events over 40 km. Family motives appear just as important, even at distances ≥100 km, with the desire to live closer to non‐resident family/friends being the most frequently cited family submotive. Estimated propensities to undertake employment and educational‐related migration fit very closely to predictions of human capital models of migration, being highest among young, residentially flexible and highly educated individuals. Migrants citing family‐related motives are disproportionately drawn from midlife and later‐life phases, with family shown to be a key motive among migrants with care‐related needs (e.g., parents with children) or access to fewer resources (e.g., social renters and low educational attainment).

## 1 INTRODUCTION

In comparison to residential mobility, which is typically thought to be associated with local‐scale family and housing‐related moves, internal migration is often defined as a (semi‐) permanent relocation taking place over a relatively long‐distance within national borders. As a macrodemographic process, internal migration can play a crucial role in affecting real changes in local area population size and composition and is thought crucial in facilitating the efficient functioning of local, regional, and national housing and labour‐markets. At the level of the individual and household, migration is generally regarded as an important and necessary mechanism for facilitating labour‐market flexibility and occupational progression and thus is often built into policies concerned with improving social mobility and regional development (Manley, Van Ham, Bailey, Simpson, & Maclennan, 2013; McCann & Ortega‐Argilés, 2015). Based on the premise that long‐distance moves are mainly motivated by work and education, and generally lead to favourable labour‐market outcomes, internal migration has long been the subject of academic and policy interest.

Microeconomic perspectives have provided important contributions to the explanation of internal migration and its interactions with human capital and labour‐market outcomes (Böheim & Taylor, 2007; Borjas, Bronars, & Trejo, 1992; Sjaastad, 1962). However, possibly as a result of the dominance of economic perspectives within migration research, the assumption that the majority of longer distance moves are labour‐market‐driven remains largely untested (though see Dixon, 2003; Niedomysl, 2011; Morrison & Clark, 2011; Geist & McManus, 2012; Clark & Maas, 2015). An obvious consequence of this is that we may be understating the significance of other motives that could have implications not just for our understanding of the drivers and patterns of internal migration but also the policies we derive from such understanding. In this article, data from the geocoded United Kingdom Household Longitudinal Study (UKHLS) are used in order to examine a range of individually stated motives, revealing how the relative importance of different motives varies according to the distance of move as well as how propensities to migrate for different reasons vary according to specific life‐course characteristics and socio‐economic conditions.

### 1.1 Migration motives and variation over distance

**Figure 1:**
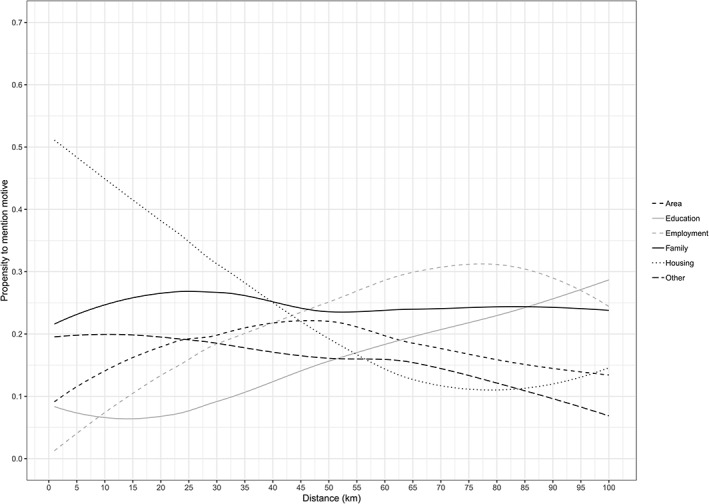
Variations in stated migration motives by distance (pooled UKHLS 2009–2016). Source: University of Essex. Institute for Social and Economic Research, NatCen Social Research, Kantar Public. (2018). Understanding Society: Waves 1–7, 2009–2016 and Harmonised BHPS: Waves 1–18, 1991–2009: Special Licence Access, Census 2001 Lower Layer Super Output Areas. [data collection]. 9th Edition. UK Data Service. SN: 6670, http://doi.org/10.5255/UKDA‐SN‐6670‐9. Estimates account for complex survey design and non‐response using the “survey” package in R (Lumley, 2018). N.B. *n* = 15,083, based on a pooled cross‐sectional sample of UKHLS wave pairs covering the period 2009–2016, includes individuals (aged 16+) who provide a motive for moving at *t*_*1*_ and move any distance between waves *t*_*0*_ and *t*_*1*_

Niedomysl's (2011) analysis of Swedish survey data provides a rare glimpse of how the primary motives for migration can change over distance. Where his analysis did find support for the common assumption linking shorter distance moves to housing and longer distance moves to employment, it also revealed how a sizable portion of long‐distance migrants are motivated by other, non‐labour‐market, factors. Despite using different measures and definitions of internal migration, the few related studies from Australia (Clark & Maas, 2015), New Zealand (Morrison & Clark, 2011), and the United States (Geist & McManus, 2012) are broadly consistent in demonstrating that, as a share of total migration events, employment‐related motives are in the minority. The only existing British‐based study of migration motives was performed by Dixon (2003), where her analysis of British Household Panel Survey data again demonstrated how the share of job‐related moves grew with distance whereas housing and partnership‐related moves declined. It should be noted however that Dixon's study used changes in geographical unit as a proxy for distance (i.e., intradistrict, interdistrict‐within‐region, and interregional), which has the undesired effect of assigning any short distance moves that cross district boundaries as longer distance migrations (a phenomenon sometimes termed “pseudo migration”). Utilising the detailed geocodes held in the UKHLS, Figure 1 provides an up‐to‐date description of how motives vary over distance in contemporary Britain.

Figure 1 indeed reveals how housing‐related motives drop off sharply with distance, whereas employment‐ and education‐related motives increase with distance. From these data, the common assumptions linking short‐distance moves to housing and longer distance moves to labour‐market considerations appear reasonable, though there are some important caveats. First, housing remains a relatively important motive for relocation even at distances of 40 km. Meanwhile, family‐related motives are shown to be of consistent significance, where even for long‐distance migration they are shown to be of a similar magnitude of importance to employment‐ and education‐related motives. Where family has often been cited in combination with housing as a key trigger for local‐scale relocation, its relevance for longer distance migration is certainly noteworthy and suggests a more nuanced understanding of those migrating over longer distances is needed.

### 1.2 Linking variations in migration motives to variations in life‐course characteristics and conditions

Since the early work of Rossi (1955), micro‐level studies have benefitted greatly from recognising how various (positive and negative) life events and transitions act as triggers for migration. Previous research would suggest that the stated motives for migrating are likely to be very closely tied to specific life‐course characteristics and conditions. Following Kley (2010), we can think of a series of broad life‐course phases in which certain motives rise to prominence as others recede. It is in *early adulthood* where the relevance of traditional economic perspectives of migration is perhaps most relevant. In almost all national contexts, migration propensities are at their peak in early adulthood, among those aged 18–25 (Fielding, 2012), with migration to university (Smith, 2009) as well as into employment (Champion, 2005) being a hallmark of the transition into independent adulthood. For migration events occurring in this period, employment and educational considerations are clearly of paramount importance, with research showing that the strongest pecuniary returns to migration are concentrated among young and highly educated migrants (Morrison & Clark, 2011). Though this is not to say that other motives will not play a role too. From the perspective of family motives, transitions into adulthood are clearly predicated on desires to gain independence and move away from family while, even if intended as a relatively temporary situation, the completion of university often coincides with the migration of graduates back to family (Stone, Berrington, & Falkingham, 2014).

Although early adulthood can be thought of as a phase of limited life “commitments” and relative (spatial) flexibility, such things as the transition into homeownership, the formation of partnerships, and the arrival of children inevitably lead to more complex decision‐making processes which involve evaluations extending far beyond maximising individual economic returns (Coulter & Scott, 2015). For those in the *family phase*, for instance, migration decision making will often include the locational balancing of two careers, with the family migration literature, and more specifically the gender‐role model, revealing female partners to be more likely to act as “tied” migrants sacrificing their own careers for the sake of their (male) partner's (see Cooke, 2008). The presence of children can add further complexity, with considerations about the potential negative impact that the changing of schools and dislocation from friendship networks can have on children's education and well‐being (Bailey, Blake, & Cooke, 2004). Such concerns presumably underpin the particularly low migration rates among families with children (Clark & Davies Withers, 2007). Yet, among the families that do migrate, migration towards members of the extended family (e.g., grandparents) can prove useful in enabling access to free and reliable supplemental childcare as well as more intensive social interaction (Silverstein & Giarrusso, 2010). Likewise, lifestyle or consumption‐based preferences, for more housing space and/or the desire for a more pleasant environment to raise children, can be key reasons motivating the decision to move (Kulu & Milewski, 2007). Beyond family formation, the breakdown of partnerships can also influence the type of migration undertaken. For instance, for separated or divorced individuals, transitions into solo‐living represent the loss of one commitment and can trigger further adjustments in housing consumption and locational preferences. Where separation and widowhood can herald a particularly vulnerable phase in the life course, previous research has hinted at the importance of non‐resident family members as an attraction factor encouraging and directing migration towards locations where family are living (Cooke, Mulder, & Thomas, 2016; Das, de Valk, & Merz, 2017). Signalling greater need, low incomes, and access to limited resources have also been associated with closer familial proximity, as well as lower propensities to move away from family and higher propensities to move towards family, as people seek low‐cost alternatives for care (Dawkins, 2006; Silverstein, 1995).

We know from the “jobs or amenities” literature that consumer amenities, lifestyle attractions, and a better climate can also be key attraction factors behind the decision to migrate (Niedomysl & Clark, 2014). In the British context, the long‐standing dominance of suburbanisation and counterurbanisation processes are often presented as evidence of the relevance of such concerns. Halfacree's (1994) detailed study of the motives behind long‐distance urban‐to‐rural migration finds environmental and housing factors to be crucial within decision‐making processes. Although suburbanisation is already present in the family phase, aggregate studies of U.K. migration flows reveal how patterns of counterurbanisation grow in strength as we move along the migrant‐age schedule (Dennett & Stillwell, 2010). Events typical to *midlife phases*, such as “empty nesting,” can underpin such patterns. Indeed, with children no longer present in the family home, the reduced demand for housing space, coupled with the loss of child‐related constraints on mobility, can prompt desires to downsize and relocate towards these amenity‐rich environments (Wulff, Champion, & Lobo, 2010).

The subsequent transition into retirement facilitates further flexibility, by removing the need for locational proximity to the workplace, with migration patterns around retirement again associated with moves down the urban hierarchy, towards rural and costal locations (Dennett & Stillwell, 2010; Fielding, 2012). It is important to recognise that such forms of amenity‐led migration are typically undertaken by individuals who have access to sufficient material (asset and/or income‐based) resources (Thomas, Stillwell, & Gould, 2015). Beyond lifestyle and environmental considerations, postretirement migration may also be influenced by the location of non‐resident family, with the presence and location of grandchildren shown to be an important attraction factor among the “young‐old” (Pettersson & Malmberg, 2009). As we move into the *late‐life phases*, frail, widowed, and less healthy people are likely to have quite specific locational needs that speak to their particular vulnerabilities (Litwak & Longino, 1987). Although the mechanisms are clearly different, closer proximity to family, and/or social and health care services, may again emerge as the dominant motives behind migration (Evandrou, Falkingham, & Green, 2010).

Despite the long‐held assumption that longer distance migration is primarily motivated by work and education, it is clear that a substantial share of internal migration is motivated by other less overtly economic motives, with family being particularly important. From the perspective of the discussion so far, this heterogeneity in motives likely reflects heterogeneity in the life‐course characteristics and conditions of contemporary populations. Thus, the analysis that follows is tasked with determining how variations in micro‐level sociodemographic and economic characteristics inform probabilities to migrate for different reasons as well as the propensity of different types of migrant to cite certain motives.

### 1.3 Data

The analysis utilises the first seven waves of the UKHLS, with (restricted access) geocodes for Census 2001 Lower Super Output Areas (LSOAs). The UKHLS contains a wealth of detailed longitudinal information on individuals and households and includes, among those who change address, the stated reasons for relocating (Knies, 2017). Critically, the size and detail of the data make it possible to provide a detailed empirical description of migration motives in contemporary Britain and an analysis of the individual and household characteristics underpinning variations in these stated reasons for migrating.

The analysis is based on a pooled cross‐sectional sample of UKHLS wave pairs covering the period 2009–2016. The use of wave pairs (*t*_*o*_ and *t*_*1*_) allows for the identification of internal migrants, their location, and their individual and household characteristics at both the origin and destination. The distance of move is calculated using the Pythagorean formula, measuring the Euclidian distance between the centroids of LSOAs at *t*_*0*_ and *t*_*1*_. LSOAs are approximate to neighbourhoods and are designed to be stable over time and consistent in size, containing a minimum of 500 and a maximum of 3,000 individuals. In cases where a move had taken place within an LSOA, a distance is derived using the intrazonal distance calculation of Batty (1976). 1Where the origin to destination distance in zone *i* is calculated as $r_{i}/\sqrt{2}$, where *r* is the radius of a circle equivalent in area (*A*) to zone *i* and where the radius can be defined as $\sqrt{A/\pi }$ (Stillwell & Thomas, 2016). Stillwell and Thomas (2016) show this to be a reasonable approximation at such detailed geographical scales.

At each wave, the UKHLS records answers to the question: “*Thinking about the reasons why you haven't lived continuously at this address since we last interviewed you, did you move from this address for …*?” Respondents are provided with six options, and the interviewer is instructed to code all that apply, the six options are family, education, employment, housing, area, and “other.” From here, each respondent is asked to provide a single specific submotive. As noted by Coulter and Scott (2015), due to data or sample size constraints, most previous studies of the motives have tended to collapse the stated reasons for moving into a limited number of broad categories. Where motives are a central focus of analysis, a substantial reduction in variable detail has the unfortunate potential to blur interesting associations and increase the likelihood of making incorrect inferences. Benefiting from the sample size and variable detail of the UKHLS, this analysis can incorporate all recorded motives, as well as a descriptive summary of stated submotives.

A wide range of individual and household variables, measured prior to any migration event (at *t*_*0*_), are collected in order to test the substantive importance of various characteristics for informing variations in the propensity to migrate for a given reason. Demographic measures include gender, age (16–24; 25–34; 35–44; 55–64; 65+), marital status (married; cohabiting couple; separated/divorced; single and never married; widowed), parental status (whether lives with dependent child or not 2A dependent child is defined as one aged under 16 or aged 16 to 18 and in school or nonadvanced further education, not married, and living with parent.), and whether the respondent is White British or not. The socio‐economic characteristics include housing tenure (homeowner; private renting; social renting), educational attainment (low, ≤GCSE; middle, post‐secondary; high, ≥BA degree), and employment status (employed, including maternity leave; full‐time student; nonemployed; retired, long‐term sick/disabled; self‐employed). A measure of respondents' health is collected and defined according to whether they have any long‐standing (12 months or more) physical or mental impairment, illness, or disability. A measure of the region at origin (for the analysis of migration propensities) and the direction of migration (for the analysis of migrants) are also included. These measures are based on the city region geography of Stillwell, Bell, Blake, Duke‐Williams, and Rees (2000), a functional geography specifically developed for the analysis of internal migration in Great Britain. The analysis is thus restricted to migration taking place within Great Britain (England, Wales, and Scotland and not Northern Ireland).

## 2 SAMPLES AND METHODS

Given that there are different ways to unpick the relationship between migration propensities, migrant characteristics, and migration motives, the analysis below is split into two parts. The first part seeks to estimate the effects of a range of sociodemographic, economic, and life‐course characteristics on the propensity to migrate for a given reason. Because motives are not mutually exclusive (i.e., respondents can give several reasons for moving), a multinomial logit model is not appropriate. Approximately 13% of migration events (at distances ≥40 km) are associated with more than one motive. Therefore, this initial analysis draws on five separate binomial logit models applied to five separate samples, one for each motive. In each sample, *y* = 1 when an individual cites a given motive after migrating a distance of ≥40 km between *t*_*0*_ and *t*_*1*_. The 40 km migration cut‐off is based on the results of Figure 1, namely, the point at which housing no longer represents the primary motive for relocation. 3Additional analyses using cut‐offs at 30 and 50 km produced similar substantive findings. When an individual migrates ≥40 km and does not cite the given motive they are censored. In order to avoid bias resulting from correlated outcomes and the double counting of stated motives, only one partner is (randomly) selected for inclusion in cases of coresidential partnerships. Each motive subsample has the same reference outcome category (*y* = 0, *n* = 221,682) of “No migration,” 4This includes short‐distance movers, that is, those who move but remain within 40 km of the origin at *t*_*0*_. which provides us with comparable estimates for the relative risk of migrating for employment (*y* = 1, *n* = 468), education (*y* = 1, *n* = 383), family (*y* = 1, *n* = 413), area (*y* = 1, *n* = 207), and housing‐related (*y* = 1, *n* = 163) reasons. Given that it is difficult to form meaningful inferences from the heterogeneous “Other” motive category (mentioned in 13% of migration events), it is not included in the analysis.

The second model‐based analysis focuses specifically on migrants, estimating the relative propensity for a migrant with a specific characteristic to report a given migration motive. This migrant‐only sample (*n* = 1,574) is restricted to those who have moved ≥40 km between *t*_*0*_ and *t*_*1*_, where again only one partner is selected in cases of coresidential partnerships. As before, five separate binomial logit models are calibrated, one for each motive, with each model estimating the propensity among migrants to mention the given motive (e.g., for employment, *y* = 1= employment motive mentioned; *y* = 0= employment motive not mentioned).

Attrition rates are higher in the UKHLS than in its predecessor, the British Household Panel Survey (Lynn & Borkowska, 2018), with migration known to be directly related to sample dropout in longitudinal studies. Subsequently, using wave *t*_*1*_ cross‐sectional weights for individual adult main interviews, differential sample non‐response, unequal selection probabilities, and sampling error are accounted for. Because the presence of respondents in the analysis is also dependent on being observed at *t*_*0*_, model‐predicted probabilities to be observed at *t*_*0*_ are used as an adjustment to the standard cross‐sectional weights. 5Based on a selection of relevant individual, household and sample characteristics, model predicted probabilities are derived via the estimation of a binomial logistic regression model (0 = not observed at *t*_*0*_; 1 = observed at *t*_*0*_) of all pooled cross‐sectional UKHLS sample respondents (Waves 2–7), that is, prior to any subsetting. Using these adjusted weights and information on survey clustering and stratification, the analysis better accounts for complex survey design and non‐response. All estimates, descriptive and model‐based, are derived using the “survey” package in R (Lumley, 2004; Lumley, 2018). An unweighted descriptive summary of the migrant‐only sample is provided in Table 1, where the relative frequencies of each motive, and the corresponding shares across each independent variable, are given.

**Table 1:** Unweighted sample frequencies and relative shares mentioning each motive: Migration ≥40 km

	Sample *n*	Employment	Education	Family	Area	Housing
Motive mentioned	1,574	0.30	0.25	0.27	0.13	0.10
Age						
16–24	645	0.27	0.52	0.11	0.05	0.06
25–34	342	0.50	0.09	0.25	0.11	0.12
35–49	235	0.37	0.09	0.35	0.18	0.10
50–64	218	0.18	0.01	0.45	0.29	0.17
65+	134	0.01	0.00	0.62	0.21	0.18
Gender						
Female	899	0.29	0.23	0.30	0.13	0.10
Male	675	0.31	0.27	0.23	0.13	0.11
Marital status						
Married	415	0.35	0.06	0.37	0.22	0.14
Cohabiting couple	159	0.33	0.11	0.30	0.18	0.15
Separated/divorced	125	0.22	0.06	0.51	0.16	0.11
Widowed	58	0.04	0.00	0.69	0.14	0.14
Single and never married	817	0.30	0.41	0.14	0.07	0.07
Lives with own child						
No	1,350	0.28	0.27	0.25	0.12	0.09
Yes	224	0.40	0.12	0.38	0.17	0.15
Housing tenure						
Homeowner	854	0.25	0.25	0.28	0.15	0.09
Private renting	581	0.42	0.24	0.20	0.09	0.12
Social renting	139	0.12	0.25	0.47	0.13	0.09
Educational attainment						
Low—≤GCSE	391	0.16	0.09	0.48	0.19	0.12
Middle—post‐secondary	570	0.20	0.49	0.18	0.08	0.08
High—≥BA degree	613	0.48	0.12	0.22	0.14	0.11
Employment status						
Employed (inc. maternity)	655	0.42	0.09	0.27	0.14	0.10
Self employed	74	0.42	0.10	0.31	0.22	0.16
Full‐time student	453	0.21	0.65	0.09	0.02	0.04
Nonemployed	182	0.36	0.13	0.34	0.15	0.14
Retired, long‐term sick/disabled	210	0.02	0.00	0.57	0.26	0.19
White British						
No	301	0.32	0.27	0.24	0.09	0.10
Yes	1,273	0.30	0.24	0.28	0.14	0.10
Long‐standing health issues						
No	1,168	0.33	0.29	0.22	0.12	0.09
Yes	406	0.21	0.13	0.41	0.16	0.13
Migration direction						
Core to core	70	0.44	0.33	0.09	0.06	0.07
Elsewhere to core	303	0.36	0.37	0.15	0.06	0.07
Core to elsewhere	235	0.24	0.25	0.28	0.18	0.18
Migration between noncore districts	966	0.29	0.20	0.32	0.14	0.10

Source: University of Essex. Institute for Social and Economic Research, NatCen Social Research, Kantar Public. (2018). Understanding Society: Waves 1–7, 2009–2016 and Harmonised BHPS: Waves 1–18, 1991–2009: Special Licence Access, Census 2001 Lower Layer Super Output Areas. [data collection]. 9th Edition. UK Data Service. SN: 6670, http://doi.org/10.5255/UKDA‐SN‐6670‐9. N.B. Sample includes only those who've moved ≥40 km with the selection of only one partner in cases of coresidential relationships.

## 3 ANALYSIS

### 3.1 Descriptive summary of migration (sub)motives

Based on the weight adjusted shares, employment (30%), education (26%), and family (25%) are the most commonly cited motives for migrating over 40 km, with area and housing‐related motives mentioned in 13% and 10% of migration events respectively. Table 2 provides the weight adjusted shares of the two most commonly cited submotives for each macromotive used in the model‐based analyses below. Over half of employment‐related migration events are motivated by the start a new job with a new employer, with only 15% linked to changes in employment within the same employer. Unsurprisingly, education‐related migration is overwhelmingly linked to moves into (58%) and out (25%) of educational establishments. From the perspective of family‐related migration, Table 2 demonstrates the importance of non‐resident family as a key attraction factor, where 53% of family‐related migration events are motivated by desires to be closer to family/friends. Moves linked to marriage and cohabitation are typically linked to short‐distance relocations (Feijten & Van Ham, 2007), though around 12% of long‐distance family‐related migration events do appear to be motivated by such factors. Just 13% of longer distance migration events are found to be motivated by area‐related considerations, 23% of which are linked to desires for a more rural environment, fitting common explanations of counterurbanisation processes. Housing‐related moves are relatively rare at distances over 40 km, though desires for more privacy and space (15%) are shown to be among the most important housing‐related reasons to migrate.

**Table 2:** Weight adjusted shares of submotives for migration: Migration ≥40 km

	Share	*SE*
Employment motives		
Moved to start a new job with a new employer	0.52	0.03
Got a different job with the same employer which meant moving workplace	0.15	0.02
Educational motives		
Moved to term‐time accommodation/college or university	0.58	0.03
Left education/ended course	0.25	0.03
Family motives		
Moved to be closer to family/friends	0.53	0.03
Married/moved in with partner	0.12	0.02
Area motives		
Wanted to move to specific place	0.29	0.04
Wanted to move to a more rural environment	0.23	0.03
Housing motives		
None of the above/Other reason	0.28	0.04
Wanted more privacy/previous accommodation overcrowded	0.15	0.03

Source: University of Essex. Institute for Social and Economic Research, NatCen Social Research, Kantar Public. (2018). Understanding Society: Waves 1–7, 2009–2016 and Harmonised BHPS: Waves 1–18, 1991–2009: Special Licence Access, Census 2001 Lower Layer Super Output Areas. [data collection]. 9th Edition. UK Data Service. SN: 6670, http://doi.org/10.5255/UKDA‐SN‐6670‐9. Estimates account for complex survey design and non‐response using the ‘survey’ package in R (Lumley, 2018). N.B. Sample includes only those who migrated ≥40 km, with the selection of only one partner in cases of coresidential relationships.

### 3.2 The propensity to migrate for a given reason

In an attempt to identify and differentiate relationships between migrant characteristics, propensities, and motives, Table 3 presents estimates for the propensity to migrate for a given reason. Table 4 then presents results of an analysis of the migrant‐only sample, which is aimed at providing insights into the differences between motivations within the group of migrants.

**Table 3:** Binomial logistic regression models for the propensity to migrate for different reasons

	Employment			Education			Family			Area			Housing		
	Coef.	*SE*	OR	Coef.	*SE*	OR	Coef.	*SE*	OR	Coef.	*SE*	OR	Coef.	*SE*	OR
Intercept	−5.97	0.35		−8.16	0.45		−5.78	0.38		−7.34	0.55		−8.05	0.53	
Gender (ref: Female)															
Male	0.13	0.12	1.14	0.11	0.14	1.12	**−0.37**	0.12	0.69	−0.04	0.17	0.96	−0.02	0.19	0.98
Age (ref: 16–24)															
25–34	**−0.41**	0.19	0.66	**−1.19**	0.30	0.31	0.05	0.27	1.05	−0.38	0.37	0.68	−0.30	0.34	0.74
35–49	**−1.40**	0.23	0.25	**−1.93**	0.35	0.15	−0.58	0.30	0.56	**−0.97**	0.39	0.38	**−0.74**	0.37	0.48
50–64	**−2.01**	0.29	0.13	**−2.77**	0.67	0.06	−0.28	0.29	0.75	−0.50	0.41	0.61	−0.43	0.42	0.65
65+	**−4.29**	1.24	0.01	**−14.89**	0.83	0.00	**−0.72**	0.34	0.49	**−1.10**	0.50	0.33	−0.70	0.56	0.50
Marital status (ref: Married)															
Cohabiting couple	**−0.89**	0.21	0.41	−0.28	0.42	0.76	−0.21	0.24	0.81	0.07	0.27	1.07	−0.11	0.34	0.89
Separated/divorced	−0.27	0.26	0.76	0.08	0.54	1.09	0.19	0.18	1.20	−0.22	0.28	0.80	−0.35	0.34	0.70
Single and never married	**−0.53**	0.17	0.59	0.05	0.35	1.05	**−0.50**	0.23	0.61	**−0.74**	0.26	0.48	−0.25	0.28	0.78
Widowed	−0.45	0.79	0.64	**−11.76**	0.43	0.00	0.14	0.22	1.15	−0.83	0.42	0.44	−0.15	0.45	0.86
Lives with own child (ref: No)															
Yes	**−0.66**	0.17	0.52	−0.40	0.29	0.67	−0.10	0.19	0.90	−0.39	0.22	0.68	−0.06	0.24	0.94
Housing tenure (ref: Homeowner)															
Private renting	**1.62**	0.13	5.06	**1.32**	0.15	3.74	**0.87**	0.14	2.39	**0.81**	0.23	2.24	**1.63**	0.22	5.09
Social renting	−0.43	0.30	0.65	0.14	0.26	1.15	0.11	0.18	1.11	−0.38	0.33	0.68	−0.31	0.40	0.73
Educational attainment (ref: Low ‐ < = GCSE)															
Middle—post‐secondary	**0.91**	0.18	2.48	**2.13**	0.24	8.42	**0.49**	0.16	1.62	**0.63**	0.22	1.89	**1.06**	0.24	2.88
High—≥BA degree	**2.29**	0.19	9.89	**2.31**	0.29	10.07	**0.63**	0.15	1.88	**1.08**	0.22	2.94	**1.30**	0.24	3.66
Employment status (ref: Employed inc. maternity leave)															
Full‐time student	**0.43**	0.17	1.53	**2.28**	0.22	9.73	0.51	0.28	1.67	−0.18	0.51	0.84	−0.04	0.39	0.96
Nonemployed	**0.48**	0.20	1.61	**0.78**	0.32	2.18	**0.51**	0.19	1.66	0.36	0.27	1.43	0.54	0.30	1.72
Retired, long‐term sick/disabled	−0.91	0.56	0.40	−0.91	1.09	0.40	**0.66**	0.19	1.93	**0.73**	0.29	2.07	0.66	0.37	1.94
Self‐employed	−0.15	0.26	0.86	0.29	0.47	1.33	0.30	0.25	1.35	0.11	0.32	1.11	0.16	0.35	1.18
White British (ref: No)															
Yes	**0.43**	0.19	1.54	**0.60**	0.19	1.82	0.03	0.20	1.03	**0.84**	0.31	2.31	**0.74**	0.30	2.11
Long‐standing health issues (ref: No)															
Yes	−0.11	0.15	0.89	−0.20	0.20	0.82	0.10	0.13	1.11	−0.20	0.18	0.82	−0.15	0.20	0.86
City region urban hierarchy (ref: Far)															
Core	**−0.99**	0.26	0.37	**−0.47**	0.23	0.63	**−0.53**	0.21	0.59	0.50	0.31	1.66	0.28	0.29	1.33
Near	−0.23	0.16	0.80	−0.07	0.20	0.94	**−0.43**	0.15	0.65	0.13	0.24	1.14	−0.32	0.25	0.72
Rest	**−0.79**	0.20	0.46	−0.39	0.21	0.67	**−0.62**	0.17	0.54	0.07	0.27	1.07	−0.45	0.29	0.64

Source: University of Essex. Institute for Social and Economic Research, NatCen Social Research, Kantar Public. (2018). Understanding Society: Waves 1–7, 2009–2016 and Harmonised BHPS: Waves 1–18, 1991–2009: Special Licence Access, Census 2001 Lower Layer Super Output Areas. [data collection]. 9th Edition. UK Data Service. SN: 6670, http://doi.org/10.5255/UKDA‐SN‐6670‐9. Estimates account for complex survey design and non‐response using the “survey” package in R (Lumley, 2018). N.B. Model fit statistics (e.g., AIC or BIC) for logit regression under complex sampling are not yet available in the “survey” package. Sample based on selection of only one partner in cases of coresidential relationships. For each outcome, migrants who cite other motives are censored. Bold values indicate statistical significance at >95% level.

**Table 4:** Binomial logistic regression models for the propensity among migrants to mention specific motives

	Employment			Education			Family			Area			Housing		
	Coef.	*SE*	OR	Coef.	*SE*	OR	Coef.	*SE*	OR	Coef.	*SE*	OR	Coef.	*SE*	OR
Intercept	−0.20	0.55		−2.05	0.73		−1.37	0.72		−4.11	1.15		−4.17	0.79	
Gender (ref: Female)															
Male	−0.03	0.15	0.97	**0.41**	0.21	1.51	−0.26	0.16	0.77	0.08	0.20	1.08	0.21	0.21	1.24
Age (ref: 16–24)															
25–34	0.11	0.23	1.12	**−0.96**	0.30	0.38	0.50	0.28	1.65	0.15	0.37	1.16	0.21	0.34	1.24
35–49	−0.30	0.29	0.74	**−0.80**	0.35	0.45	0.61	0.31	1.85	0.54	0.40	1.71	0.37	0.41	1.44
50–64	**−1.14**	0.36	0.32	**−1.99**	0.75	0.14	**1.20**	0.33	3.32	**0.92**	**0.41**	**2.51**	**1.02**	0.47	2.78
65+	**−3.24**	1.21	0.04	**−14.70**	0.69	0.00	**1.39**	0.45	4.03	0.34	0.59	1.40	1.16	0.67	3.20
Marital status (ref: Married)															
Cohabiting couple	**−0.75**	0.29	0.47	0.12	0.46	1.13	0.29	0.30	1.34	0.45	0.32	1.57	**0.84**	0.38	2.31
Separated/divorced	−0.54	0.37	0.58	−0.09	0.59	0.92	0.43	0.26	1.53	−0.22	0.31	0.81	−0.10	0.40	0.91
Single and never married	**−0.53**	0.24	0.59	0.69	0.39	2.00	−0.24	0.27	0.78	−0.24	0.29	0.78	0.53	0.32	1.70
Widowed	0.06	1.01	1.06	**−13.52**	0.83	0.00	0.41	0.38	1.51	**−0.93**	0.51	0.40	−0.09	0.53	0.91
Lives with own child (ref: No)															
Yes	−0.35	0.23	0.71	0.62	0.36	1.86	**0.53**	0.24	1.70	0.22	0.29	1.25	**0.88**	0.33	2.41
Housing tenure (ref: Homeowner)															
Private renting	**0.52**	0.18	1.68	−0.32	0.22	0.73	−0.27	0.19	0.77	**−0.50**	0.24	0.60	**0.48**	0.24	1.62
Social renting	−0.19	0.35	0.83	−0.21	0.34	0.81	**0.65**	0.26	1.91	−0.63	0.41	0.53	−0.35	0.42	0.70
Educational attainment (ref: Low ‐ < = GCSE)															
Middle—post‐secondary	−0.20	0.23	0.82	**1.12**	0.28	3.06	**−0.42**	0.20	0.66	−0.29	0.25	0.75	**0.57**	0.28	1.77
High—≥BA degree	**0.97**	0.22	2.64	0.40	0.30	1.49	**−0.89**	0.20	0.41	−0.25	0.25	0.78	0.07	0.25	1.07
Employment status (ref: Employed inc. maternity leave)															
Full‐time student	**−0.83**	0.22	0.44	**2.02**	0.24	7.51	**−0.58**	0.27	0.56	**−1.52**	0.49	0.22	**−1.34**	0.39	0.26
Nonemployed	−0.06	0.24	0.94	0.42	0.34	1.52	0.35	0.22	1.42	−0.46	0.31	0.63	0.18	0.31	1.20
Retired, long‐term sick/disabled	**−1.42**	0.61	0.24	**−14.39**	0.44	0.00	0.23	0.32	1.25	0.47	0.36	1.59	0.63	0.51	1.88
Self‐employed	0.38	0.31	1.46	0.85	0.48	2.33	−0.02	0.31	0.98	0.10	0.37	1.10	0.14	0.43	1.15
White British (ref: No)															
Yes	0.23	0.21	1.26	−0.35	0.27	0.70	−0.21	0.21	0.81	0.57	0.32	1.78	0.20	0.29	1.22
Long‐standing health issues (ref: No)															
Yes	−0.09	0.18	0.92	−0.39	0.26	0.68	0.11	0.17	1.12	−0.19	0.21	0.83	0.04	0.23	1.04
Migration direction (ref: Core to Core)															
Core to noncore	**−0.99**	0.45	0.37	−0.33	0.65	0.72	0.46	0.61	1.58	**2.69**	1.07	14.72	**1.28**	0.66	3.60
Noncore to core	0.05	0.45	1.05	−0.43	0.65	0.65	0.03	0.62	1.03	**1.81**	1.08	6.11	0.59	0.70	1.81
Migration between noncore districts	−0.38	0.42	0.69	−0.39	0.62	0.68	0.58	0.59	1.79	**1.98**	1.04	7.24	0.29	0.65	1.34

Source: University of Essex. Institute for Social and Economic Research, NatCen Social Research, Kantar Public. (2018). Understanding Society: Waves 1–7, 2009–2016 and Harmonised BHPS: Waves 1–18, 1991–2009: Special Licence Access, Census 2001 Lower Layer Super Output Areas. [data collection]. 9th Edition. UK Data Service. SN: 6670, http://doi.org/10.5255/UKDA‐SN‐6670‐9. N.B. Model fit statistics (e.g., AIC or BIC) for logit regression under complex sampling are not yet available in the “survey” package. Sample includes only those who've moved ≥40 km with the selection of only one partner in cases of coresidential relationships. Estimates account for complex survey design and non‐response using the “survey” package in R (Lumley, 2018). Bold values indicate statistical significance at >95% level.

For employment‐led migration, the results in Table 3 fit very closely to what would be expected from human capital models of migration (e.g., Sjaastad, 1962), with the youngest and most educated in the population having the highest propensities to migrate for employment‐related reasons. Indeed, along with the higher relative propensities observed for the nonemployed and full‐time students (at t_0_), such characteristics are typical of those who stand to gain the most from migration in terms of employment, occupational progression and/or income (Morrison & Clark, 2011). Perhaps unsurprisingly, one's location also appears important in shaping the likelihood of migrating for employment, where the highest relative propensities for employment‐led migration are found among those residing in regions farthest from urban cores, where migration up the urban hierarchy can be expected to be more conducive to employment or occupational progression. As noted above, the arrival of various alternative life “commitments” is likely to introduce complexity into decision‐making processes and thus shift evaluations of whether, when and where to migrate far beyond simple attempts to maximise economic returns (Coulter & Scott, 2015). The results in Table 3 offer some support to this supposition, with the propensity to migrate for employment approximately 50% lower among resident parents than nonparents and approximately five times higher among private renters than homeowners. Interestingly, although there is sure to be a lot of variation between ethnic minority groups, the propensity to migrate for employment is estimated to be about 1.5 times higher for the White British population than the minority ethnic population.

According to the size of the coefficients in Table 3, the propensity to migrate for education appears to be even more heavily stratified by age and educational attainment. Unsurprisingly, given the micromotives in Table 2, the highest propensities to migrate for education are found among those with typical characteristics of individuals moving to and from university: young adults, with entry‐level (A‐level) or post‐university (≥BA degree) educations, in the private rental sector, working outside of the labour‐market (either nonemployed or students). Moreover, given that most universities are located in Britain's larger towns and cities, we see the propensity to migrate for education reduces as we move up the urban hierarchy, from “Far” to “Core” regions. Again, it should be noted that the propensity to undertake long‐distance education‐led migration is almost two times higher among the White British population than among the ethnic minority population.

Moving away from the more overtly labour‐market‐oriented forms of migration, the results in Table 3 show how the strength of the typical age and educational gradients to migration are reduced. This is particularly the case for family‐led migration where, having accounted for a range of socio‐economic characteristics, the propensity to migrate for family appears fairly even across the age groups with the only pronounced difference being between those in the very youngest (16–24) and very oldest (65+) age groups. Migrating for family, area, and housing is clearly more common among the retired and long‐term sick/disabled, than those in employment, which presumably reflects their disconnect from labour‐market‐oriented concerns. Where the presence of children in the home is shown to reduce the likelihood of migrating for employment, the deterring effect of resident children on migration is not observed in the context of family migration or housing‐led migration. As noted above, migration towards members of the extended family can be attractive in offering the potential for free and reliable supplemental childcare, whereas the desire for more housing space is also known to motivate families to move (Kulu & Milewski, 2007). Where previous research suggests that women have stronger relationships with family and tend to be more engaged in support exchange than men (Klein Ikkink, Van Tilburg, & Knipscheer, 1999; Rossi & Rossi, 1990), the results show men to be less likely to migrate for family reasons than women. It is also notable that the typically higher propensity to migrate among the White British majority disappears in the context of family‐led migration. Finally, although frail and less healthy people were assumed to have specific needs that might increase their likelihood of migrating towards family or towards certain areas with social and health care services, long‐standing health issues appear to have little influence on the propensity to migrate, regardless of motivation.

### 3.3 Differences between motivations within the group of migrants

Although the results in Table 3 are useful in demonstrating the relative risks of migrating for different reasons, the considerable effect of variations in educational attainment, age, and housing tenure can obscure differences between motivations within the group of migrants. For instance, although overall propensities to migrate reduce with age, some subtle variations emerge when we focus only on those who do migrate. Indeed, according to the results in Table 4, the youngest and most educated migrants are shown to be disproportionately motivated by employment and educationally led factors, whereas migrants citing housing, family, and area‐related motives are disproportionately drawn from more established populations, reflecting age groups we typically associate with family forming, midlife, and later‐life phases.

Similarly, although we know that lower educational attainment is associated with lower overall propensities to migrate (Table 3), the results presented in Table 4 demonstrate how migrants with lower educational attainment are considerably more likely to cite family‐related concerns than those with high educational attainment. The same pattern is observed for migrants with children, who are estimated to be 1.7 times more likely to cite a family‐related motive than those without, and migrants living in the subsidised social housing sector, who are almost twice as likely to cite family‐related reasons than homeowners. Again, from the perspective that family networks are important sources of informal support and care, these observations would fit with the argument that those with fewer resources (e.g., social renters and those with lower levels of human capital), or those with particular needs (e.g., parents with dependent children), are more likely to seek closer proximity to family than would otherwise be the case (Dawkins, 2006; Silverstein, 1995). Meanwhile, as compared with their reference groups, home‐owning migrants, migrants moving from core to noncore regions, and those around retirement age appear disproportionately likely to cite area‐related motives. Fitting with the area‐related submotives recorded in Table 2, these characteristics indeed typify those we associate with amenity‐led suburbanisation and urban–rural shift (Dennett & Stillwell, 2010; Fielding, 2012). Among migrants citing housing‐related motives, quite a small group at this distance, higher relative propensities are observed among those in the preretirement ages and among parents. This, combined with higher rates for migrants moving from core to noncore regions, also fits with what we would expect in cases of family suburbanisation (Kulu & Milewski, 2007). Meanwhile, reflecting more “flexible” life‐course characteristics, migrants in cohabiting couples (as compared with married) and in the private rental sector (as compared with homeowners) also have higher relative propensities to cite housing‐related motives. Analysis of this migrant‐only sample further demonstrates the limited influence of long‐standing health issues for influencing recorded reasons for moving, whereas variations among the group of migrants according to ethnic minority status are limited too.

## 4 CONCLUSION

Where internal migration is typically assumed to be motivated by employment and educational opportunities, and more local‐scale residential mobility by housing and family considerations, the results of this analysis suggest the reality is far more nuanced. Certainly, an increase in the distance of migration is associated with an increase in the propensity to cite employment and educational‐related motives as well as a decline in the propensity to cite housing‐related motives. However, family motives are also associated with a considerable share of longer‐distance migration events, representing approximately 25% of moves at distances of 40 km or more—a share similar to that of employment and education. A descriptive summary of the submotives reveals that the most frequent family‐related migration motive is based around the desire to live closer to family/friends (representing 53% of all family‐related migration events).

Fitting with human capital models of migration, model‐based analyses reveal that the youngest and most educated in the population, as well as full‐time students and the nonemployed, are the most likely to migrate for employment‐related reasons. Likewise, individuals living farthest from urban cores, and thus farthest from the most dynamic labour‐markets, are also more likely to migrate for employment related reasons than those already living in major urban centres. Taken together, all such characteristics are typical people who could be expected to gain the most from labour‐market migration in terms of improving occupational progression, gaining employment and/or achieving greater pecuniary returns. Migration for education is also heavily focused on those in the youngest age groups, with the highest propensities linked to characteristics typical of pre‐university and post‐university student migration—for example, entry‐level (A‐level) or post‐university (≥BA degree) qualifications, private renters, the nonemployed, or students.

Although family is of similar relevance to employment and education as a motive for longer distance migration, it appears quite different in its composition. Regardless of the motivation, the propensity to migrate is always higher among the younger and more educated population, and those living in the “flexible” private rental sector. However, migrants citing family‐related motives are disproportionately drawn from midlife and later‐life phases, whereas the arrival of various life commitments and needs also appears to increase the propensity to cite less overtly economic motives for moving. For instance, although the presence of dependent children in the household tends to be associated with lower overall propensities to migrate, the deterring effect of children on migration is not found in the case of family‐related migration. Using a migrant‐only sample, it is also shown that migrants with children are disproportionately likely to have cited family as a motive for their move. A similar pattern emerges for those living in subsidised social housing as well as those with the lowest levels of educational attainment, suggesting that family is a particularly important motive among individuals with care‐related needs or access to fewer resources. It also appears that migration for family is more common among women than men.

Although housing and area‐related motives were less commonly cited, the characteristics associated with them fit closely to what would be expected in the British context. Indeed, fitting with long‐standing patterns of amenity‐led urban–rural shift, home‐owning migrants, migrants moving from core to noncore regions, and those around retirement age are particularly likely to cite area‐related motives. Meanwhile, higher relative propensities to cite housing‐related motives are observed among migrants with children and those moving away from urban core regions, which again fits with patterns typical of well documented processes of family suburbanisation.

Given that analyses of the role of non‐resident family networks in internal migration still rare, future research in this area could hold great potential in providing new insights into why, when, and where people migrate. Following the results presented here, ongoing macroprocesses such as population ageing, persistent welfare‐state retrenchment, and the rising importance of informal familial systems within social care (Pavolini & Ranci, 2008) could be expected to further increase the significance of non‐resident family as a motivating factor for migration. Studies examining how the importance of different motives changes over time would thus not only prove interesting but also potentially crucial as we seek to more accurately explain migration behaviours or predict migration flows and patterns. The same can be said of the need for similar analyses in different national contexts, where differing population structures, economic and housing geographies, welfare regimes, and familial traditions may all contribute to different findings from those presented here on the British context. With several suitably detailed large scale geocoded surveys already in existence in different national contexts—for example, the Household, Income and Labour Dynamics in Australia (HILDA), the German Socio‐Economic Panel (SOEP), and the Panel Study of Income Dynamics (PSID) in the United States—comparable analyses should be pursued in an attempt to better inform academics and policymakers of the real, rather than assumed, motives underpinning migration in contemporary societies.
